# Mathematics–gender stereotype endorsement influences mathematics anxiety, self‐concept, and performance differently in men and women

**DOI:** 10.1111/nyas.14779

**Published:** 2022-04-16

**Authors:** Serena Rossi, Iro Xenidou‐Dervou, Emine Simsek, Christina Artemenko, Gabriella Daroczy, Hans‐Christoph Nuerk, Krzysztof Cipora

**Affiliations:** ^1^ Centre for Mathematical Cognition Loughborough University Loughborough United Kingdom; ^2^ Talim ve Terbiye Kurulu Baskanligi MEB, Ankara Turkey; ^3^ Department of Psychology University of Tübingen Tübingen Germany; ^4^ LEAD Graduate School & Research Network University of Tübingen Tübingen Germany

**Keywords:** arithmetic performance, mathematics anxiety, mathematics self‐concept, gender stereotype endorsement, gender differences, structural equation modeling

## Abstract

Mathematics anxiety (MA) is negatively associated with mathematics performance. Although some aspects, such as mathematics self‐concept (M self‐concept), seem to modulate this association, the underlying mechanism is still unclear. In addition, the false gender stereotype that women are worse than men in mathematics can have a detrimental effect on women. The role that the endorsement of this stereotype (mathematics–gender stereotype (MGS) endorsement) can play may differ between men and women. In this study, we investigated how MA and mathematics self‐concept relate to arithmetic performance when considering one's MGS endorsement and gender in a large sample (*n* = 923) of university students. Using a structural equation modeling approach, we found that MA and mathematics self‐concept mediated the effect of MGS endorsement in both men and women. For women, MGS endorsement increased their MA level, while in men, it had the opposite effect (albeit weak). Specifically, in men, MGS endorsement influenced the level of the numerical components of MA, but, unlike women, it also positively influenced their mathematics self‐concept. Moreover, men and women perceived the questions included in the considered instruments differently, implying that the scores obtained in these questionnaires may not be directly comparable between genders, which has even broader theoretical and methodological implications for MA research.

## Graphical abstract

In this study, we investigated how MA and mathematics self‐concept relate to arithmetic performance when considering one’s mathematics–gender stereotype endorsement and gender in a large sample (*n* = 923) of university students. We found that MA and mathematics self‐concept mediated the effect of MGS endorsement in both men and women.

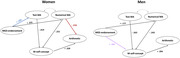

## Introduction

Research in mathematics, so far, has mainly focused on the cognitive underpinnings of mathematics difficulties. However, besides cognitive abilities, other aspects seem to play a role. These include emotional aspects, such as mathematics anxiety (MA),^1^ personal beliefs, such as mathematics self‐concept (hereafter, M self‐concept),^2^ but also societal influences, such as gender stereotypes toward mathematics.^3^, ^4^

Gender is a crucial aspect to consider when investigating factors that can influence mathematics performance. Women tend to have higher levels of negative emotional feelings, such as higher MA,^5^, ^6^, ^7^, ^8^ and lower levels of positive self‐beliefs, such as a lower M self‐concept.^9^, ^10^ Moreover, despite its falsity (there are no systematic gender differences in mathematics performance),^7^, ^11^ there is a prevalent misconception that mathematics is for men, and not for women, also called a mathematics–gender stereotype.^12^, ^13^ All these aspects might contribute to women being under‐represented in the mathematics‐intensive STEM (science, technology, engineering, and mathematics) field,^14^ across countries.^15^ To this regard, the endorsement (agreement) of the mathematics–gender stereotype may have an even more important role, but so far, little is known about its role on MA, M‐self‐concept, and arithmetic performance in university students. The present large‐scale study set out to address this issue by examining how one's MA and M self‐concept are related to arithmetic performance when considering mathematics–gender stereotype endorsement and gender.

### Mathematics anxiety

MA is among the most thoroughly investigated affective factors influencing mathematical performance.^1^, ^16^ It is defined as “a feeling of tension and anxiety that interferes with the manipulation of numbers and the solving of mathematical problems in […] ordinary life and academic situations*”* (p. 551).^17^ MA is a specific form of anxiety that is related to, but distinct from, other forms of anxiety, such as trait, social, and test anxiety.^8^, ^18^ MA can be further differentiated into the anxiety of being evaluated in mathematics, anxiety of evaluation and examination settings (mathematics test anxiety), and anxiety associated with use of mathematics in everyday life situations (numerical anxiety).^19^

Overall, MA is negatively associated with mathematics performance (*r ≈* −0.30).^20^, ^21^ Nevertheless, the underlying causal mechanism between MA and mathematics performance is still debated.^22^ The relationship between MA and performance may be bidirectional, which materializes as a vicious circle: poor mathematics performance can trigger MA in some individuals, and MA, in turn, can further reduce their mathematics performance.^22^, ^23^ This vicious circle often functions differently than for other anxieties and phobias. As a negative emotional response to situations involving mathematics, MA can lead to stress and avoidance behavior.^24^ While a vicious circle in these cases is presumably mainly maintained by avoidance and inner reward for that, for many people, mathematics cannot be avoided on an educational or professional level. Thus, it is essential to understand the mechanism that underlies the influence of MA on mathematics performance. Relevant factors include an individual's characteristics, such as their M self‐concept,^25^, ^26^ as well as societal and contextual factors,^27^ such as the level of endorsement of mathematics–gender stereotypes.^3^, ^4^

### Mathematics self‐concept

Mathematics self‐concept is defined as one's beliefs about their competence in mathematics (e.g., “I am good at mathematics”), or their beliefs to which they are a mathematics person.^2^, ^28^ In a broader sense, self‐concept is defined as a person's self‐perception in a certain domain.^29^ Importantly, M self‐concept is distinct from mathematics self‐efficacy,^30^ which is the belief in one's capacity to execute a mathematics task (e.g., “I can do this mathematics problem”).^31^, ^32^ Mathematics self‐concept is positively related to mathematics performance (*r ≈* 0.50)^25^ and conversely, it is negatively related to MA (*r ≈* −0.70).^26^ Despite this strong relationship, M self‐concept and MA are separate and empirically distinguished from each other.^30^ On the one hand, low M self‐concept makes an individual feel less capable of handling environmental requests (e.g., difficult mathematics tasks) increasing MA as a consequence. On the other hand, experiences of high MA can distort one's self‐perception, leading to the belief that a person is unable to solve mathematics tasks, and thus to a lower M self‐concept.^33^ Thus, M self‐concept might be a mediator in the relationship between MA and mathematics performance.^34^ Justicia‐Galiano and colleagues^34^ verified this assumption in primary‐school children; however, little is known about the role of M self‐concept in adults. Specifically, anxiety becomes more differentiated during development, and MA increases due to the increase in the difficulty of mathematics being taught and the effects of the aforementioned vicious cycle.^1^, ^35^, ^36^ Consequently, the potential mediational role of the M self‐concept in this relationship could be different in adults compared to children.

### Gender differences in mathematics anxiety and mathematics self‐concept

Although women perform comparably to men in mathematics,^7^, ^11^ there are robust gender effects in MA and M self‐concept. Women often report higher MA than men in adulthood,^5^, ^6^ adolescence,^7^ and primary school age.^8^ Also, women often report a lower M self‐concept compared to their same‐ability male peers during precollege and college years.^9^, ^10^ However, most of such differences have been observed using self‐reports, which do not necessarily reflect the true underlying constructs.

Differences in the scores of self‐report measures between genders can, on the one hand, reflect actual differences in levels of measured constructs, or they may be due to women tending to be more open to reporting anxiety than men. This can originate from men's greater search for social desirability and thus a lower propensity to express their emotions than women.^1^

Furthermore, most MA and M self‐concept studies assume that men and women conceptualize and interpret questions similarly, without first checking whether this is statistically warranted; in other words, measurement invariance across genders is taken for granted. Additionally, gender differences might also originate from broader societal beliefs and expectations, such as gender stereotypes.^37^, ^38^

### Mathematics–gender stereotype and its endorsement

Mathematics–gender stereotype is the false idea that mathematics is for men, not for women.^12^, ^13^ A stereotype is a cognitive link between two social or personal concepts that are not defining features for one another.^39^, ^40^ Thus, stereotyping is the application of a stereotype, inferring the characteristic of one thing from the characteristic of another thing.^40^ Through the mechanism of stereotype threat, when a stereotype is made salient or relevant for the task at hand, members of a stereotyped group may be susceptible to confirming the negative stereotype of their ingroup (their own group).^41^ Regarding gender, women are often influenced by stereotype threat in different activities,^42^, ^43^ including mathematics.^3^, ^4^ This means that women tend to perform worse than men right after being reminded of the negative stereotype that women are worse than men in this particular activity (but see Refs. 38, 44, and 45 for failed replications of stereotype threat studies in different domains).

Beyond immediate performance impairment, a stereotype can also have a long‐term effect.^46^ To this regard, stereotype endorsement, that is, the level of agreement with a stereotype, plays a crucial role.^47^ Specifically, mathematics–gender stereotype endorsement (MGS endorsement) regards the degree of agreement with or endorsement of this stereotype.^48^ However, its effect on mathematics performance and related emotional aspects have not been thoroughly studied.

A stereotype can be endorsed by both the group being stereotyped (here women) and the group which is not stereotyped (here men). According to Tajfel,^49^ the identity of an individual is based on their membership in social groups. Generally, individuals have a natural tendency to show favoritism to their own group, and, therefore, the non‐stereotyped group should easily endorse a negative stereotype regarding its outgroup (i.e., men likely to endorse MGS about women). Although members of the stereotyped group should not endorse the stereotype, sometimes they end up approving status stereotypes regarding their ingroup (i.e., women endorsing the MGS).^50^ Therefore, the effect that MGS endorsement can have on other mathematics‐related aspects is most likely different between the stereotyped group (women) and the non‐stereotyped group (men). Moreover, the nature of the mathematics–gender stereotype is different in relation to an individual's self in women and men. In female students, MGS endorsement might be a predictor of negative attitudes toward mathematics and potentially of lower involvement in mathematics‐related professions.^51^ For instance, women may believe themselves to be generally more mathematics anxious than they actually are while facing mathematics problems.^50^ In contrast, in male students, higher MGS‐endorsement is related to higher self‐perceptions in mathematics as reported by Bieg and colleagues.^52^ These findings point to potential differential effects of MGS endorsement in men and women.

### Pending questions

With a notable exception of Bieg and colleagues’ study,^52^ research on MGS‐endorsement has mainly focused on its effect on women (stereotyped group), while less is known of its effect on the nonstereotyped group (men). Also, research so far has not investigated the role of MGS endorsement on MA, M self‐concept, and arithmetic performance concurrently and its potential effects in men and women. When investigating the relationship between some of these aspects, previous studies mainly used multiple regression models, which have several limitations: among them is the crucial assumption that variables are measured without error. This assumption is particularly problematic because observed variables in psychosocial subjects, such as the present constructs of interest, are measured with a non‐negligible proportion of error. To overcome this issue, we used structural equation modeling (SEM), where we can account for measurement error on both the measurement and observed level.^53^ Moreover, SEMs allow us to validly and accurately investigate the direct and indirect effects, as well as mediations between the constructs of interest.^53^

Some previous studies found measurement invariance between men and women in MA and M self‐concept,^54^, ^55^, ^56^, ^57^ although such analyses are relatively scarce. Even less is known about measurement invariance across gender in MGS endorsement. Moreover, we cannot be sure that MA and M self‐concept are interpreted in the same way across gender without explicitly verifying it in our sample.

### The present study

**Figure 1:**
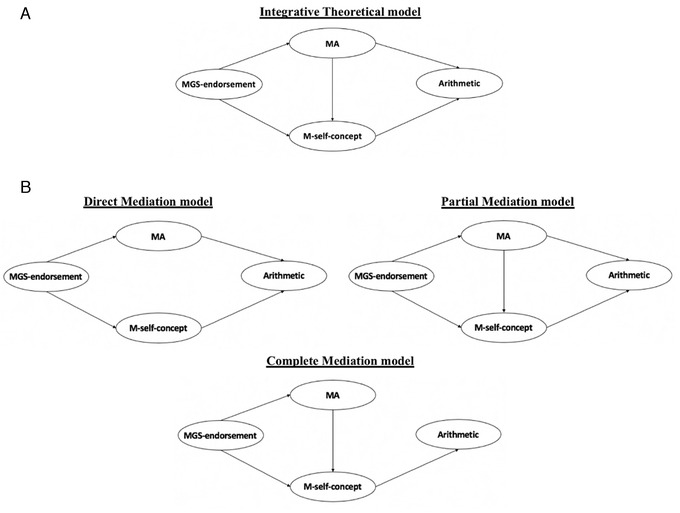
(A) The integrative theoretical model association between mathematics–gender stereotype endorsement (MGS endorsement), mathematics anxiety (MA), mathematics self‐concept (M self‐concept), and arithmetic performance (Arithmetic). (B) The compared nested mediation models between mathematics–gender stereotype endorsement (MGS endorsement), mathematics anxiety (MA), mathematics self‐concept (M self‐concept), and arithmetic performance (Arithmetic)

This study aimed to investigate (1) the relationships between MGS endorsement, MA, and M self‐concept, (2) how they concurrently influence university students’ arithmetic performance (Fig. 1A), and (3) how gender influences these relationships.

As a performance measure, we assessed students’ arithmetic performance because it is one of the basic mathematics skills acquired and developed over childhood and adolescence and widely used in daily life throughout adulthood.^58^ Moreover, there are large individual differences in arithmetic performance not only in children but also in adults.^59^ Even though some university students have deliberately chosen not to pursue a mathematics‐related degree, mathematics and especially arithmetic is involved in many other subjects (e.g., geography, psychology, and fine arts).^60^ Therefore, it is crucial to understand how university students’ arithmetic performance can be influenced by their individual characteristics, societal and contextual factors, and the associated gender differences, as arithmetic skills can be advantageous for gaining their degree and functioning outside the academic context.

We addressed the following research questions. (1) Are MA, M self‐concept, MGS endorsement, and arithmetic interpreted in the same manner by men and women? (2) Does a measurement model comprising of MGS endorsement, MA, M self‐concept, and arithmetic performance obtain a good model fit in both men and women; that is, does the empirical structure of the constructs reflect the theoretical one? (3) Is there a difference in the structural predictive pattern between the variables in question between men and women; specifically, does MGS endorsement influence MA, M self‐concept, and arithmetic performance; does M self‐concept mediate the link between MA and arithmetic performance; are there gender differences in these relationships?

We predicted that in each considered construct, the overall factor structure would be the same for men and women and that MGS endorsement, MA, M self‐concept, and arithmetic performance would be related in both gender groups as there was no clear evidence in the literature that this is not the case.

The potential different structural patterns between the constructs were investigated as per our preregistration (see https://osf.io/5erxv). We predicted that MGS endorsement directly influences both MA and M self‐concept. We further predicted a mediational role of M self‐concept in the relationship between MA and arithmetic performance. Specifically, we predicted one of the following patterns of results (Fig. 1B):

1. Both MA and M self‐concept directly influence arithmetic^52^ (direct mediation model).
2. MA influences M self‐concept and both influence arithmetic performance (partial mediation model).
3. MA influences M self‐concept, which in turn influences arithmetic performance^34^ (complete mediation model).

Regarding gender differences, we predicted that MGS endorsement will have a different effect on men and women. For women, we predicted MGS endorsement to have a disruptive influence on the other constructs,^51^, ^61^ but less is known about its effects on men's MA, M self‐concept, and arithmetic performance. Therefore, we hypothesized that MGS endorsement will not influence the other three constructs in men, since they are not the stereotyped group in this study.

## Method

This study was a secondary data analysis preregistered in the Open Science Framework (OSF) (https://osf.io/5erxv). Data came from a large‐scale online survey, conducted at the University of Tuebingen in Germany. Data analyses differ slightly from those reported in the preregistration. Specifically, in the preregistration, we forgot to mention the first step in SEM, which regards the identification of a good baseline model for each construct. This step was included in the present data analyses.

### Participants

Participants were university students at the University of Tübingen, recruited via university e‐mails and social networks. An initial sample of 1285 participants completed the online survey. Participants were excluded if they were not German speakers (*n* = 33); reported an age above 100 (*n* = 1; probably a dishonest response); and were not university students (*n* = 200: 7 pupils, 12 apprenticeships, 152 employers, and 29 other). Only German native speakers were eligible to ensure proper understanding of relatively complex and nuanced questionnaire items. Furthermore, we excluded participants who did not complete the arithmetic task in the given order, as indicated in the instructions (*n* = 128). Instead, they skipped some of the items during task completion. We excluded them because this task had a time limit, and items were mixed on the basis of operation types and complexity. Thus, by skipping complex items and only solving the simple ones, a participant may have scored higher than they would have if they solved the problems in the presented order. The final sample included 923 participants (629 women and 294 men; age: *M* = 22.8 years, SD = 3.9). Among them, 45.3% were undertaking a degree without any mathematics courses, 41.4% were undertaking a degree with some mathematics courses, and 13.3% were undertaking a degree with mostly mathematics courses. The study was approved by the ethics committee of Medical Faculty of the University of Tübingen.

### Materials

During the online survey, which lasted about 15 min, participants were presented with a timed arithmetic task, which was followed by three self‐report questionnaires. The instruments are described below in the order of their administration.

#### Arithmetic task

A speeded calculation task was composed of 40 arithmetic problems, including four basic operations (addition, subtraction, multiplication, and division) to be completed with a time limit of 2 minutes. The problems were presented in a fixed randomized order and participants were instructed to complete them in the given order (without skipping any items). Each operation was represented by 10 problems divided into simple and complex categories, based on the complexity (carry/non‐carry for additions, borrow/non‐borrow for subtractions, and part of the multiplication table up to 10/above 10 for multiplications and divisions). The addition and subtraction problems included two two‐digit operands or one three‐digit and one two‐digit operands. The total score was the sum of items solved correctly, thus a high score corresponded to high performance. This task shows good reliability (Cronbach's α = 0.92; 0.93 and 0.91 for men and women, respectively).

#### Mathematics self‐concept

The mathematical ability subscale of the German adaptation^62^ of the Self‐Description Questionnaire (SDQ) III^63^ was used to investigate participants’ M self‐concept. The scale comprises four statements regarding ability in mathematics (e.g., “I am good in math.”). Participants were asked to indicate on a 4‐point Likert scale to which extent they agreed with the statements. The total score was calculated by summing up responses to all items (item 2 and item 4 were reverse‐coded), thus a higher score corresponded to a higher level of M self‐concept. This measure showed good reliability (Cronbach's α = 0.90: 0.88 and 0.90 for men and women, respectively).

#### Mathematics anxiety

MA was measured with the Mathematics Anxiety Rating Scale‐Short questionnaire (MARS‐Short),^64^ translated into German for this study. It comprises 30 items, divided into two subscales (math test anxiety and numerical anxiety*)*. Each item describes mathematics‐related situations that may lead to anxiety. Participants had to indicate on a 5‐point Likert scale how anxious they would feel in each of those situations. The total score is calculated by summing up responses to all items of the mathematics test anxiety subscale (the first 15 items) and to all items of numerical anxiety subscale (the last 15 items). A higher score corresponded to a higher level of MA. This measure showed good reliability (Cronbach's α = 0.95; 0.94 and 0.95 for men and women, respectively).

#### Mathematics–gender stereotype endorsement

MGS endorsement was examined with the male domain scale of the Fennema–Sherman Mathematics Attitudes Scale‐Short Form questionnaire (FSMAS‐SF),^65^ translated into German for this study. The scale is composed of nine statements, some of which concern the stereotype stating that mathematics is a male domain and men are better in this subject than women, while others state that mathematics is not a gender domain and women are as good in mathematics as men. The total score was calculated by summing up responses to all items (items 1, 2, 3, and 4 were reverse‐coded), so a higher score corresponded to a greater endorsement of the stereotype that mathematics is a male subject. This measure showed good reliability (Cronbach's α = 0.80; 0.81 and 0.79 for men and women, respectively).

### Procedure

The data were collected using the SoSci Survey^66^ online software. After providing informed consent, participants were asked for demographic information (age, gender, and first language) and details about their educational background (highest educational qualification), current occupation, and/or field of study. Subsequently, the following data were collected: arithmetic performance task, M self‐concept questionnaire, MA questionnaire, and MGS endorsement questionnaire. Except for the arithmetic task, no time constraints were forced, so each participant completed the items in their own time. The entire data collection lasted 56 days.

### Statistical analysis

First, we obtained descriptive statistics for each construct, zero‐order correlations between them separately for men and women and checked item distribution for normality using R statistical software. Measurement models (confirmatory factor analysis; CFA), measurement invariance, and SEMs were run using Mplus 8.1.^67^

**Figure 2:**
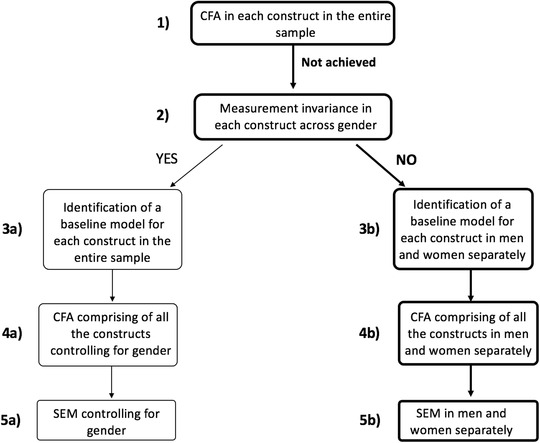
Data analyses decision tree. The bolded path b is the one we followed

The decision tree of the subsequent data analyses steps is reported in Figure 2. CFA for each construct in the entire sample was conducted to verify their structure reliability (Fig. 2, step 1). The model tested for each construct (MGS endorsement, MA, M self‐concept, and arithmetic performance) was chosen based on the existing literature.^62^, ^64^, ^68^ The measurement model of MGS endorsement included a single latent variable with the nine items in the questionnaire as indicators. For the M self‐concept, the measurement model included a single latent variable and each of the four items were indicators. For MA, we specified a measurement model, which included MA as a latent variable and each of the 30 items in the presented questionnaire were indicators (MA1 model). We also tested a measurement model that comprised of the two different subscales of the MA questionnaire, namely, mathematics test anxiety (test MA) and numerical anxiety (numerical MA), as latent variables and the items for each subscale were corresponding indicators (MA2 model). Finally, the measurement model of arithmetic performance included a single latent variable with the composite scores of each different type of operation (addition, subtraction, multiplication, and division) as indicators.

Measurement invariance (see Fig. 2, step 2) allows us to investigate whether each of the considered constructs is measured alike in men and women. In other words, measurement invariance tests whether scores from an instrument that assesses an unobserved construct (e.g., MA, M self‐concept, and MGS endorsement) reflect the same meaning under different conditions (i.e., over two populations, in our case in men and women).^53^ There are different steps to test measurement invariance: configural, metric, scalar, and strict invariance. If configural invariance is achieved, it would mean that the overall factor structure is similar in men and women. In that case, we can proceed with testing metric invariance, which measures if the factor loadings are equivalent across the two groups. If this last step achieves good data fit, we can proceed with testing scalar invariance, in which both the factor loadings and the items’ intercepts would be the same in the two groups. In addition to these steps, we could also test strict invariance, in which also the residual variances of items would be equal across gender. However, strict invariance does not have to be achieved to claim measurement invariance. If scalar invariance is achieved, the two groups can be compared on their scores in the latent variables.^69^ As seen in Figure 2, if measurement invariance is achieved in all the constructs, the two groups can be compared on the basis of their scores in the latent variables. In that case, we would run the next set of analyses by controlling for gender (moderator analysis; path a). On the other hand, if measurement invariance is not achieved in each construct, the scores in the latent variables cannot be directly compared between the two groups. Consequently, if the latent scores cannot be directly compared, the same is true for the relationships between them. This indicates that we should examine men and women separately in the subsequent CFA and SEM analyses (path b).^69^

Model fit was assessed according to well‐known cutoff criteria.^70^, ^71^ These include CFI (comparative fit index) and the TLI (Tucker–Lewis index) that need to be close to 0.95 or greater (≥ 0.90 for acceptable fit), the RMSEA (root mean square error of approximation), and the SRMR (standardized root mean residual) that need to be close to 0.05 or smaller to demonstrate good fit to the data (≤ 0.10 for acceptable fit).

When checking the items’ distribution for normality in each construct, departures from normality of some of the items were found (skewness and kurtosis range outside the values of −1 and 1)^72^ (see sections 2S and 3S in Supplementary Materials at OSF https://doi.org/10.17605/OSF.IO/2JMFK for skewness, kurtosis, and frequency distribution of every single item in each considered measure), therefore, the maximum likelihood mean‐adjusted estimator was used in the models. It provides the Satorra–Bentler scaled chi‐square value (SB*χ* ^2^),^73^ and so nested models in measurement invariance and SEM analyses were compared using Satorra–Bentler scaled chi‐square difference tests ΔSB*χ* ^2^ and the related *P* values.

## Results

### Descriptive statistics and correlations

Table 1 reports the descriptive statistics (means, standard deviations, minimum and maximum values, skewness, and kurtosis) of each considered construct separately for each gender.

**Table 1:** Descriptive statistics of the four considered constructs for men and women

		*N*	*M*	SD	Min	Max	Skewness	Kurtosis
Men	MGS endorsement	294	13.82	5.30	9	41	1.75	3.75
	MA	294	59.01	17.43	30	118	0.66	0.25
	M self‐concept	294	11.96	3.21	4	16	−0.51	−0.82
	Arithmetic	294	15.86	7.26	1	40	0.75	1.00
Women	MGS endorsement	629	12.45	4.31	9	33	1.76	3.19
	MA	629	68.73	20.24	30	149	0.50	0.07
	M self‐concept	629	10.75	3.54	4	16	−0.22	−1.06
	Arithmetic	629	11.77	5.93	1	39	0.63	1.01

MGS endorsement, mathematics–gender stereotype endorsement; MA, mathematics anxiety; M self‐concept, mathematics self‐concept; Arithmetic, arithmetic performance; *M*, mean; SD, standard deviation.

Section 1S in Supplementary Materials at OSF (https://doi.org/10.17605/OSF.IO/2JMFK) shows zero‐order Pearson correlations between the considered constructs separately for each gender. For instance, in women, MGS endorsement correlated positively with MA and negatively with M self‐concept, while no significant correlation was found with arithmetic performance. On the contrary, in men, no significant correlations were found between MGS endorsement and the other constructs. Given that we did not find a significant correlation between MGS endorsement and arithmetic performance in men or women, we did not include the direct arrow from the two constructs in our models, and we hypothesized an indirect effect of MGS endorsement on arithmetic performance (see Fig. 1).

### CFAs for each construct in the entire sample

CFAs for each construct were conducted to verify the latent factor structure of each construct in the entire sample using underlying hypothetical structures from the literature (Fig. 2, step 1). The fit indices for each construct are reported in Table 2A. Except for arithmetic performance, which demonstrated good fit indices, the other constructs did not show adequate fit to the data.

**Table 2:** (A) Fit indices of the measurement model for each considered construct in the entire sample (see step 1 in Fig. 2), and (B) in each gender after having added suggested modification indices (see step 3b in Fig. 2)

A	Construct	CFI	TLI	RMSEA	SRMR
Entire sample	MGS endorsement	0.861	0.814	0.075	0.054
	M self‐concept	0.967	0.900	0.203	0.030
	MA1	0.611	0.583	0.128	0.116
	MA2	0.788	0.772	0.094	0.209
	Arithmetic	1.00	1.00	0.000	0.002

B	Construct	CFI	TLI	RMSEA	SRMR
Men	MGS endorsement	0.980	0.967	0.040	0.042
	M self‐concept	0.995	0.973	0.099	0.009
	MA1	0.899	0.880	0.065	0.104
	MA2	0.931	0.922	0.052	0.058
	Arithmetic	1.00	1.00	0.000	0.002
Women	MGS endorsement	0.962	0.943	0.036	0.036
	M self‐concept	1.00	0.997	0.035	0.003
	MA1	0.905	0.887	0.095	0.067
	MA2	0.948	0.941	0.049	0.053
	Arithmetic	1.00	1.00	0.000	0.002

MGS endorsement, mathematics–gender stereotype endorsement; MA, mathematics anxiety; M self‐concept, mathematics self‐concept; Arithmetic, arithmetic performance.

### Measurement invariance

Since most of the measurement models of the considered constructs did not demonstrate a good fit to the data in the entire sample, measurement invariance across gender was tested in each construct to investigate whether they were measured alike in women and men (Fig. 2, step 2). Measurement invariance for MGS endorsement did not hold; configural invariance indices demonstrated a poor fit to the data and was rejected based on the absolute goodness of fit indices (CFI = 0.855, TLI = 0.807, RMSEA = 0.077 [90% CI = 0.066, 0.089], SRMR = 0.059). Similarly, in M self‐concept and both MA models, configural measurement invariance was not found (M self‐concept: CFI = 0.963, TLI = 0.888, RMSEA = 0.214 [90% CI = 0.176, 0.253], SRMR = 0.030; MA1: CFI = 0.602, TLI = 0.572, RMSEA = 0.128 [90% CI = 0.125, 0.131], SRMR = 0.120; MA2: CFI = 0.794, TLI = 0.779, RMSEA = 0.092 [90% CI = 0.089, 0.095], SRMR = 0.113). Regarding arithmetic performance, configural invariance indices showed a good fit to the data (CFI = 1.00, TLI = 1.00, RMSEA = 0.000 [90% CI = 0.000, 0.038], SRMR = 0.002), as well as metric invariance (CFI = 0.998, TLI = 0.997, RMSEA = 0.042 [90% CI = 0.000, 0.079], SRMR = 0.031). The comparison between the two nested models (metric versus configural) for arithmetic performance demonstrated that the configural model had a better fit than the metric one (ΔSB*χ* ^2^ *= *103.292, *P* < 0.05) (see Section 4S in Supplementary Materials at OSF (https://doi.org/10.17605/OSF.IO/2JMFK)).

### Baseline models for each construct in each gender

**Figure 3:**
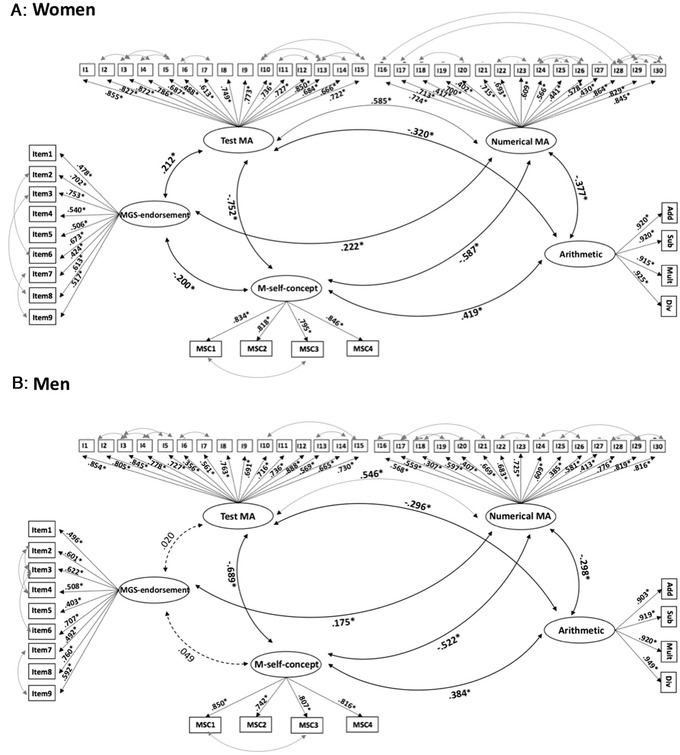
(A and B) CFAs measurement models of mathematics–gender stereotype endorsement (MGS endorsement), the two MA components (test MA and numerical MA), mathematics self‐concept (M self‐concept), and arithmetic performance in women, A, and men, B. ^*^ *P* < 0.001. black lines correspond to significant relationships between constructs, while dashed lines correspond to nonsignificant relationships between constructs

**Figure 4:**
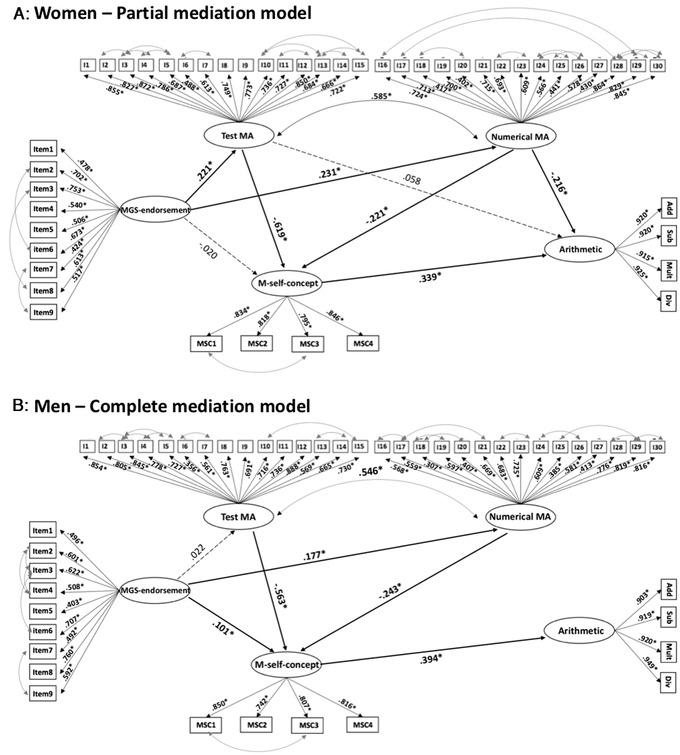
Best‐fitting SEM models. (A and B) Partial mediation model in women, A, and complete mediation model in men, B. ^*^ *P* < 0.001. Black lines correspond to significant influence of a construct on another construct, while dashed lines correspond to nonsignificant influence of a construct on another construct

Measurement invariance results indicated that scores obtained for MA, M self‐concept, and MGS endorsement did not have the same meaning for men and women. Therefore, CFA and SEM analyses should be run separately in men and women (see Fig. 2, path b). To do this, an acceptable baseline model for men and one for women for each construct were identified. For each construct, a model with the latent variable of the respective items was run. Since these models did not show adequate fit to our data, suggested modification indices^67^, ^74^ (slightly different for men and women) were consulted. These modification indices indicated some strong covariances between indicators/items, which means strong correlations between items within the same scale (see double‐arrow curved lines between indicators in Figs. 3 and 4). We accepted these modification indices (one at a time) because the items were similar in content within them, and, therefore, covariance between them was theoretically acceptable.^74^ The fit indices of the baseline models in each gender found for each construct are reported in Table 2B. Regarding MA, the model that achieved a good fit to the data was the one with the two questionnaire subscales (test MA and numerical MA) as latent variables and the corresponding items as indicators. For all the constructs, all observed variables loaded onto the respective latent variables, and all factor loadings were significant (*P* < 0.001).

### CFA with all the constructs for each gender

Using the identified baseline models, CFAs for each gender, including all the constructs, were run to determine the latent factor structure of all the constructs assessed. The CFAs showed a good fit to the data (CFI = 0.948, TLI = 0.944, RMSEA = 0.037 [90% CI = 0.034, 0.040], SRMR = 0.050) in women (Fig. 3A), as well as in men (CFI = 0.934, TLI = 0.929, RMSEA = 0.042 [90% CI = 0.038, 0.046], SRMR = 0.057) (Fig. 3B). These two measurement models were used to examine the structural association of the latent variables (SEM), respectively, for each gender.

### Structural equation models

#### Women

Three SEM models were analyzed (direct mediation model, partial mediation model, and complete mediation model; see Fig. 1B and hypotheses above for a pictorial and detailed description). The fit indices obtained for each model in each gender are reported in Table 3A.

**Table 3:** (A) Fit indices, and information needed for performing the Satorra–Bentler scaled chi square difference test of the three nested models tested, respectively, in women and men; (B) comparison between models using the Satorra–Bentler scaled chi‐square difference test

A								
Women								
Model	RMSEA	CFI	TLI	SRMR	Free parameters	Chi square	df	Scaling factor
+	0.044 (0.041–0.046)	0.928	0.922	0.131	172	2209.512	1003	1.1836
Partial	0.037 (0.035–0.040)	0.948	0.944	0.050	174	1869.387	1001	1.1831
Complete	0.037 (0.035–0.040)	0.947	0.943	0.052	172	1884.377	1003	1.1827

Men								
Model	RMSEA	CFI	TLI	SRMR	Free parameters	Chi square	df	Scaling factor
Direct	0.047 (0.043–0.051)	0.917	0.910	0.126	171	1660.919	1004	1.1158
Partial	0.042 (0.038–0.046)	0.934	0.929	0.057	173	1519.569	1002	1.1145
Complete	0.042 (0.038–0.046)	0.934	0.929	0.058	171	1522.828	1004	1.1144

B					
Women					
	Cd	TRd	Ddf	*P* value	
Direct versus partial	1.4338	281.4148	2	< 0.001	Partial better than direct
Complete versus partial	0.9825	17.2834	2	< 0.001	Partial better than complete

Men					
	Cd	TRd	Ddf	*P* value	
Direct versus partial	1.7671	90.3705	2	<0.001	Partial better than direct
Complete versus partial	1.0643	3.2696	2	0.1950	Complete better than partial

Cd, difference test scaling correction; TRd, Satorra–Bentler scaled chi‐square difference test; Ddf, difference between degrees of freedom of the two models; *P* value, *P* value of the Satorra–Bentler scaled chi‐square difference test.

The direct mediation model demonstrated a poor fit to the data. All structural path coefficients were significant (*P* ≤ 0.001; βs ranging from −0.244 to 0.279), with the exception of the path between the test MA component and arithmetic performance (β = −0.008; *P* = 0.847). The partial mediation model demonstrated a better fit to the data. Some of the structural path coefficients were significant (*P* ≤ 0.001; βs ranging from −0.619 to 0.339), with the exception of the paths between MGSE endorsement and M self‐concept (β = −0.020, *P* = 0.435) and between test MA and arithmetic performance (β = 0.058, *P* = 0.293). Finally, the complete mediation model showed a good fit to the data. All structural path coefficients were significant (*P* ≤ 0.001; βs ranging from −0.613 to 0.433), with the exception of the path between MGS endorsement and M self‐concept (β = −0.019, *P* = 0.470).

ΔSB*χ* ^2^ for nested models was conducted to compare the different nested models (Table 3B). The partial mediation model fit the data significantly better than the complete mediation model (ΔSB*χ* ^2^ *= *17.283, *P* < 0.001). The comparison between the direct model with the partial model showed that the latter fits the data significantly better (ΔSB*χ* ^2^ *= *281.415, *P* < 0.001), demonstrating that for women the partial model was the one that best fit our data (Fig. 4A). With this model structure, 20.7% (SE = 0.028, *P* < 0.001) of the variance of arithmetic performance was explained in women. The variance explained in test MA, numerical MA, and M self‐concept was 4.9% (SE = 0.014, *P* < 0.001), 5.3% (SE = 0.019, *P* < 0.05), and 60% (SE = 0.027, *P* < 0.001).

#### Men

Similarly, we ran the three SEM models for men (Table 3A). The direct mediation model demonstrated a poor fit to the data. All the structural path coefficients were significant (*P* < 0.05; βs ranging from −0.148 to 0.271), except for the path between MGS endorsement and test MA (β = 0.017; *P* = 0.256), the path between MGS endorsement and M self‐concept (β = 0.029; *P* = 0.498), and the one between test MA and arithmetic performance (β = −0.066; *P* = 0.285). The partial mediation model showed a better fit to the data. All structural path coefficients were significant (*P* < 0.05; βs ranging from −0.564 to 0.306), except for the paths between MGS endorsement and test MA (β = 0.022, *P* = 0.749) and between test MA and arithmetic performance (β = −0.0149, *P* = 0.848). Finally, the complete mediation model showed an adequate fit to the data. All structural path coefficients were significant (*P* < 0.05; βs ranging from −0.563 to 0.394), except for the path between MGS endorsement and test MA (β = 0.022, *P* = 0.749).

ΔSB*χ* ^2^ tests for nested models were conducted (Table 3B) to compare the direct mediation model with the partial mediation model. The partial mediation model fit the data significantly better than the direct mediation model (ΔSB*χ* ^2^ *= *90.371, *P* < 0.001). However, the comparison between the complete mediation model with the partial mediation one showed that the latter did not fit the data significantly better (ΔSB*χ* ^2^ *= *3.269, *P* = 0.195), demonstrating that for men the complete mediation model was the one that best fit our data (Fig. 4B). With this model structure, a moderate amount of variance (15.5%; SE = 0.040, *P* < 0.001) in arithmetic performance was explained in men. The variance explained in M self‐concept was much larger (52.2%; SE = 0.044, *P* < 0.001).

## Discussion

Our study aimed to investigate the interactive relationship between mathematics–gender stereotype endorsement, MA, mathematics self‐concept, and arithmetic performance in university students. Past studies investigated the relationship between only two of these constructs concurrently (e.g., simple correlations of MA with performance,^20^, ^21^ or MA with M self‐concept,^26^ or M self‐concept with performance,^25^ or MGS endorsement with MA^52^). This study is the first to investigate gender effects on the relationship between all these constructs concurrently, and their interactive effect on arithmetic performance. Moreover, although gender differences in MA^5^, ^6^, ^7^, ^8^ and M self‐concept^9^, ^10^ are well established, little is known about the role that endorsement of mathematics–gender stereotypes has in men and women. This study addressed these issues by investigating a large sample using a rigorous analytical approach: SEM.

First, we examined whether each construct was interpreted conceptually in the same way by men and women. Despite our expectations, measurement invariance across gender revealed that except for arithmetic performance, in all the other three constructs (MGS endorsement, M self‐concept, and MA) the overall factor structure was not the same across men and women (even the first step of measurement invariance, the configural invariance, was not achieved). This means that women and men perceived the questions posed in the considered self‐report questionnaires differently, and, therefore, a comparison of their scores would not be reliable.^69^ Our findings were unexpected, especially for MA and M self‐concept. Indeed, some previous studies investigated and found measurement invariance across gender in MA in different age groups and languages;^54^, ^55^ however, they used a different instrument (the Abbreviated Math Anxiety Scale; AMAS^75^). Although measurement invariance was found in the German version of the MARS‐short questionnaire (the same version we used) by Pletzer *et al*.,^56^ to the best of our knowledge, there are no other previous studies that investigated measurement invariance in the German version of this questionnaire. Differences between our study and Pletzer *et al*.^56^ could be explained by differences in sample characteristics: Pletzer *et al*.^56^ adapted the MARS‐Short questionnaire in the German language with university students in Austria,^56^ while our sample consisted of students in Germany. At the time Pletzer's data were collected, the universities in Austria had less stringent admission requirements compared to German universities when we collected our data. However, this tentative explanation requires investigation in future studies. The cultural and linguistic differences between participants in previous studies compared to ours may explain the non‐measurement invariance we found for M self‐concept. Measurement invariance across gender has been found testing M self‐concept in English with English speakers,^57^ while, to the best of our knowledge, there are no previous studies that tested it in German. The result regarding MGS endorsement is less surprising, given that the questions posed to the participants are strongly gender‐related (e.g., “It is hard to believe a female could be a genius in mathematics,” “I would have more faith in an answer for a math problem solved by a man than a woman”), compared to MA and M self‐concept questionnaires. Indeed, in the MA and M self‐concept questionnaires, gender is not named at all, while for answering MGS endorsement questions, people must explicitly come to terms with the gender they belong to and respond based on that, thinking about their ingroup and outgroup. Therefore, it stands to reason that men and women would see and consider these questions differently. The failure to achieve measurement invariance indicated that we must analyze the data from men and women separately.

SEMs demonstrated that MGS endorsement influenced arithmetic performance through different patterns of mediation in the two genders. In women, results showed a partial mediation role of M self‐concept in the relationship between MGS endorsement, the two components of MA (mathematics test anxiety and numerical anxiety), and arithmetic performance. This means that our data suggest that women's MGS endorsement affects both the MA components, these influence their M self‐concept, which in turn then influences their arithmetic performance. However, it was only a partial mediation as their arithmetic performance was not only positively influenced by their M self‐concept but also negatively influenced by numerical MA.

**Figure 5:**
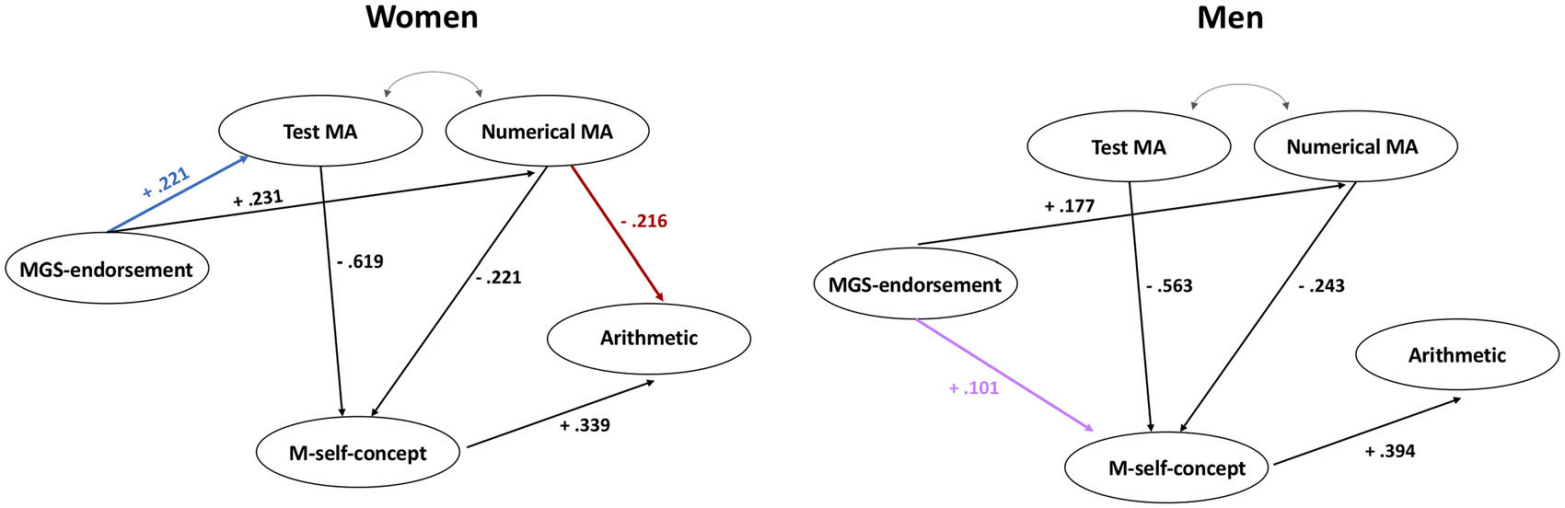
Differences in the significant structural predictive pattern between constructs in women and men. Blue arrow (MGS endorsement test MA in women) indicates effect existing in women but not in men; red arrow (numerical MA arithmetic in women) indicates another effect existing in women but not in men, while purple arrow (MGS endorsement M self‐concept in men) indicates an effect existing in men but not in women

On the other hand, in men, we found that the complete mediation of M self‐concept in the relationship between MGS endorsement, the two MA components (mathematics test anxiety and numerical anxiety), and arithmetic performance best fit the data. This means that MGS endorsement had a positive (albeit weak) influence on their levels of M self‐concept and numerical MA, and their M self‐concept influenced their arithmetic performance. It was a complete mediation because, on the one hand, both MA components affected their M self‐concept (as for women), which in turn influenced their arithmetic performance. On the other hand, unlike women, their arithmetic performance was not directly influenced by their MA components (see Fig. 5 for a comparison of the results between women and men). These results highlight some important issues.

First of all, it is well known that women tend to show higher levels of MA compared to men,^5^, ^6^, ^7^, ^8^ and that among the different causes of this gender difference, there is also the presence of gender stereotypes toward mathematics.^76^ Our study demonstrated that the level of endorsement of this gender stereotype in women plays a crucial role and may further elucidate some underlying mechanisms. Continuous exposure to negative mathematics–gender stereotypes throughout childhood could lead girls to accept and endorse them over time, and this would then influence their MA and M self‐concept, with negative consequences also on mathematics performance. Our findings are partly consistent with those by Bieg and colleagues.^52^ They observed that women, who endorsed the mathematics–gender stereotype and had low M self‐concept, reported higher MA at the trait level than the actual anxiety they experienced when solving the mathematics task. Our model, in turn, revealed that, for women, the mere presence of MGS endorsement (without a concurrent low level of M self‐concept) may be enough to raise their level of (trait) MA, which then triggers a ripple effect on the other constructs (M self‐concept and arithmetic performance). In addition, the literature has reported that mathematics anxious individuals tend to take fewer mathematics courses, avoid, where possible, additional mathematics classes, and get lower grades in those they do attend.^77^ Therefore, given the impact that MGS endorsement has on MA in women in our study, we can assume that MGS endorsement is one of the possible causes for which women are under‐represented in the mathematics‐intensive STEM fields. Indeed, MGS endorsement could contribute to increasing the trait MA in women, who may tend to avoid mathematics and, therefore, a mathematics‐related career. It is important to note that this claim should be further verified in longitudinal studies.

Beyond the negative influence that MGS endorsement can have on women, we also found that it seemed to be both positive and negative for men. Specifically, MGS endorsement had a positive (albeit weak) effect on their M self‐concept. However, we also found a weak disruptive effect of MGS endorsement on men's numerical MA. One of the possible explanations for this result could be that by endorsing the stereotype that they should be better than women in mathematics, their self‐demands increase, and this can trigger some MA. Nevertheless, as we will explain later, our results seem to show that, among the two MA components, the only MA component affected by MGS endorsement in men (the numerical one) is less relevant than the test MA one in the relationships we found. Therefore, the influence that MGS endorsement has on MA in men should not be as disruptive as it is for women (with the MGS endorsement influencing both MA components). Moreover, although the positive influence of MGS endorsement on the level of men's M self‐concept was quite weak, this is not present in women and so we can hypothesize that such stereotypes work more in favor of men and, therefore, have a slight advantage over women.

Second, in our model, we also tested the role of M self‐concept in the relationship between MA and arithmetic performance. As previously observed by Justicia‐Galiano and colleagues^34^ in primary school children, we also found a mediational role of M self‐concept in the relationship between MA and arithmetic performance in men and women, albeit in women, the mediation of M self‐concept was only partial and not complete as in men. Although our findings seem to agree with the Justicia‐Galiano and colleagues’ result,^34^ it is worth noting that the substantial methodological differences between our study and theirs do not allow for easy, direct comparison. Justicia‐Galiano *et al*.^34^ tested primary school children, who, as already explained earlier, are likely to be different from adult students due to their shorter experiences in educational settings. Furthermore, they also tested their working memory capacity, while we did not consider any general cognitive measure. Finally, they analyzed the data using simple regression, which can have some limitations, while we used SEM, which is more accurate and precise.

As previously mentioned, the components of MA, test MA, and numerical MA played different roles in our models. While MA in both genders influenced M self‐concept, test MA seemed to play a more prominent role in defining the level of M self‐concept compared to numerical MA (test MA: women β = −0.619, men β = −0.563; numerical MA: women β = −0.221, men β = −0.243). This suggests that the fear of undertaking a mathematics test can potentially have a stronger effect on mathematics performance than the fear of using mathematics in everyday situations. This outcome is in line with some authors who have claimed that, in the MARS questionnaire, the test MA component plays a primary role in the definition of the overall level of MA, while the numerical MA component is less relevant.^78^, ^79^ However, in our case, a possible explanation for this higher importance of the test MA component could be that for the population we considered (university students), test situations are more relevant in comparison to everyday arithmetic problems, since they are still performing exams in an academic context. Nevertheless, for women, we found a negative and direct effect of the numerical MA component on arithmetic performance, while there was no such direct effect for the test MA, where the effect was fully mediated by M self‐concept. In any case, the mediated effect of test MA on arithmetic performance was stronger than the one of numerical MA.

Although this study contains insightful findings, it also has some limitations. First, it lacks a more general cognitive measure. For instance, working memory is strongly involved in mathematics achievement, in particular in the acquisition of arithmetic skills and in the execution of mathematics problems.^80^, ^81^, ^82^ More specifically, mental arithmetic skills seem to rely on the phonological loop (storing information temporarily) and the central executive (e.g., when carrying out procedures) components of working memory (WM).^83^ Moreover, working memory plays a crucial role in the relationship between MA and mathematics performance.^84^, ^85^ Therefore, it would be interesting to investigate how an individual's WM capacity can mitigate or strengthen our results. Second, we did not consider other forms of anxiety, such as general anxiety and test anxiety, which can also have an impact on mathematics performance,^86^, ^87^ and mediate the relations between the constructs considered in this study. Future research should investigate whether, and how, general cognitive abilities and other forms of anxiety moderate the effect of the found relationships in a broader context. Third, we measured MGS endorsement using an explicit self‐report questionnaire (the male domain subscale of the FSMAS‐SF questionnaire).^65^ More in general, we used self‐report measures to assess all our constructs. As already mentioned, self‐reports can be biased, due to social desirability and other factors. Therefore, future research should further investigate these aspects, for instance, using measures beyond self‐reports. Fourth, the study was conducted online so that the environment was not as controlled as in a laboratory study. However, the survey was completely anonymous, participants did not have any social pressure and did not receive a reward for their performance, which makes cheating less likely. Finally, we did not assess performance, or gender stereotype endorsement, in other domains besides mathematics, therefore, we cannot generalize our results or claim that the effect of MGS endorsement is specific to mathematics. Although the lack of discriminant validity measures can be seen as a limitation of our study, it could also be viewed as a strength. Our results show how endorsement of the stereotype can be related to anxiety and performance within the domain of mathematics. Future research should clarify if our findings are specific only to mathematics‐related aspects or if they can be further generalized over other domains.

In summary, our study demonstrated that MGS endorsement can have a negative effect on women since it can increase their levels of MA, which in turn affects their level of M self‐concept and, therefore, their arithmetic performance. On the contrary, in men, MGS endorsement seems to play a slightly positive role that enhances (albeit weakly) their level of M self‐concept and, in turn, their performance. For this reason, we assume that MGS endorsement could partially explain the gender differences in the mathematics‐related emotional aspects, and eventually the under‐representation of women in mathematics‐intensive fields. Although MGS endorsement cannot be considered the only responsible factor, our study shows that we should increase the awareness of its role.

Given the effect that MGS endorsement seems to have on adult students, future research should investigate it from a developmental perspective. Specifically, it would be relevant to understand at which age and in which contexts children or adolescents begin to endorse the gender stereotype toward mathematics. Beilock and colleagues^88^ conducted a study with primary‐school children and found that their teachers’ MA could trigger and enhance girls’ endorsement of the belief that boys are better than girls in mathematics (MGS endorsement). Therefore, another important aspect to be investigated is the origin of students’ MGS endorsement. That could help researchers develop an intervention for promoting educational practices, which would avoid the triggering of mathematics–gender stereotypes at school. This could potentially put a stop to the cascading effect that MGS endorsement can have in the educational setting and future career choices.

Finally, unexpectedly, we found that women and men did not conceptually interpret the questions posed in the administered self‐report measures conceptually in a similar manner. This has methodological implications for the MA research field. Many previous studies compared the level of MA between men and women, taking for granted that the considered self‐report was interpreted in the same manner across genders. However, the comparison between groups in a specific score/level is only reliable if measurement invariance of the considered instrument has been achieved across those groups.^69^ This observation is valid not only for MA but also for M self‐concept and MGS endorsement. Therefore, our study demonstrates that it is always important to check for measurement invariance, especially when investigating gender‐related emotional and personal aspects, even when using standardized measures.

## Competing interests

The authors declare no competing interests.

### Peer review

The peer review history for this article is available at: https://publons.com/publon/10.1111/nyas.14779
